# Effects of ultrasound-assisted extraction and transglutaminase treatment on the physicochemical properties of protein from *Stropharia rugosoannulata*

**DOI:** 10.1016/j.ultsonch.2025.107637

**Published:** 2025-10-21

**Authors:** Lingyu Yang, Lili Yang, Xi Feng, Yang Xiao, Xiaoju Tian, Wen Huang, Ying Liu

**Affiliations:** a College of Food Science and Technology, Huazhong Agricultural University, Wuhan, Hubei 430070, China; b Alternative Protein Technology Innovation Center of Hubei Province, Wuhan, Hubei 43000, China; c Department of Nutrition, Food Science and Packaging, San Jose State University, San Jose, CA 95192, United States; d Institute of Applied Mycology, Huazhong Agricultural University, Wuhan, Hubei 430070, China; e School of Food Science and Engineering, Ningxia University, Yinchuan, Ningxia 750021, China; f Yinchuan Yibaisheng Bio-engineering Co., Ltd., Yinchuan, Ningxia 750002, China

**Keywords:** Ultrasonic, *Stropharia rugosoannulata* protein, Transglutaminase, Modification, Functional properties

## Abstract

Protein from edible mushrooms can serve as a new alternative protein to replace meat in various food formulations. However, the processing characteristics of mushroom protein were poor. In this study, ultrasound-assisted extraction (UAE) and transglutaminase (TGase) were used for the modification of protein from *Stropharia rugosoannulata* (SRP) to improve its utilization. The optimal parameters of UAE were 480 W for 20 min. The process of TGase modification was optimized by a single-factor experiment and response surface test to obtain the TGase dosage of 0.36 % (w/w), cross-linking at 40℃ for 1.5 h, and reaction pH of 5.4. The effects of UAE and TGase treatment on the structure and functional properties of SRP were further investigated. UAE could improve the protein yield, the water and oil-holding capacity of SRP. The SRP was cleaved into small molecular fragments, and the protein particle size was decreased. The levels of β-sheet decreased, and the random coil increased by the UAE treatment. The water-holding capacity, oil-holding capacity, and emulsifying properties of SRP increased after the modification of TGase due to the cross-linking action. The results indicated that UAE and TGase treatment could alter the structure of SRP and improve its functional properties. Our results suggested that the UAE and TGase modification technology would be applied to the utilization of SRP as alternative protein for plant-based meat analogs in the future.

## Graphical abstract

**Figure:**
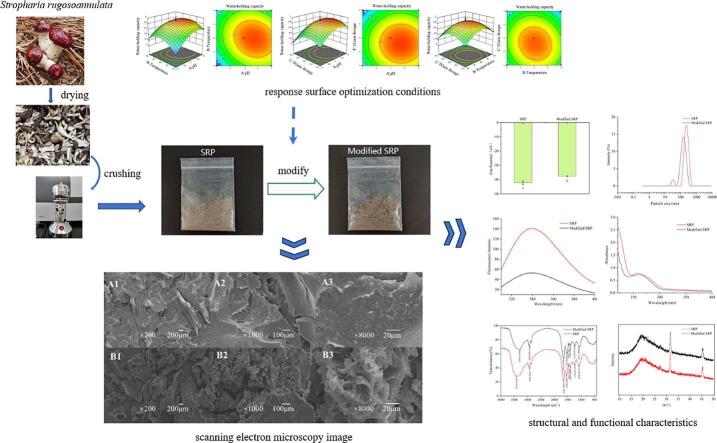

## 1 Introduction

In recent years, plant protein has been used as a functional nutrient to replace meat protein in various food formulations due to its high nutritional, cost-effective, ethical, sustainable, and health values [1]. Edible mushrooms have been recognized as a promising new source of protein, not only for their high protein content, but also for their multiple unique physiological activities (antioxidant, antiviral, immunomodulatory, etc.) [2]. *Stropharia rugosoannulata* is a well-known edible mushroom recommended by the Food and Agriculture Organization of the United Nations (FAO). *S. rugosoannulata* is a special-flavored edible mushroom that contains a large amount of protein, peptides, and amino acids. Its protein content is as high as 25.75–34.17 %, which is 1.8 times, 1.4 times, and 1.3 times that of *Agaricus bisporus*, *Lentinula edodes*, and *Pleurotus ostreatus*, respectively [3]. The *S. rugosoannulata* protein (SRP) has potential to be used as a high-quality alternative protein resource. However, SRP prepared by conventional methods has poor functional properties and processing characteristics, which limit its development and utilization in the food industry. Therefore, to explore potential future applications, further studies on the extraction, structural, and functional characterization of SRP are necessary.

Currently, the traditional protein extraction method is mainly alkali-soluble acid precipitation method, which have a low extraction rate, a long extraction time, and are prone to changes in the structure and properties of active substances. UAE technology is a new type of non-thermal green technology, which is widely used in the extraction of edible mushroom proteins because of short time consumption, simple process, low solvent dosage, and low environmental pollution [4]. It can destroy the cell walls of raw materials and increase tissue permeability through cavitation effects [5], enhancing protein extraction rates while improving functional properties by regulating protein particle size, secondary structure, and solubility [6]. This approach has demonstrated advantages in protein extraction from kidney beans, soybeans [7], and millet [8], but its systematic application in SRP extraction remains underdeveloped.

Protein modification technology is one of the methods to improve the choke point of protein processing characteristics [9]. Currently, the techniques of protein modification primarily include physical, chemical, and enzymatic methods. Physical modification is safe and non-polluting, but its modification effect has certain limitations. Although chemical modification has obvious effects, it usually requires the addition of chemical substances in the reaction process, which can produce waste and cause safety problems. Compared to physical and chemical modification, enzymatic modification is widely used in protein modification due to its mild reaction conditions, high safety, high specificity, no harmful substances, and low cost [10,11]. Transglutaminase (TGase) is a commercially available food-grade crosslinking enzyme that can be used to crosslink plant proteins with a wider selection of proteins and better cross-linking properties than commonly used modified enzymes [12,13]. TGase treatment method is an efficient, green, and safe protein modification method, and its influence on altering protein structures has been widely studied [[14], [15], [16]]. The application on edible mushroom proteins is limited, and there has not yet been any research report on the direct modification of SRP to improve its functional properties.

Therefore, this study focused on the core issue of SRP processing characteristics, and applied ultrasonic and transglutaminase treatment to extract and modify SRP. The changes in functional characteristics and protein molecular structure before and after modification were analyzed. Our results would offer guidance for a broad utilization, industrial manufacturing, and premium deep processing of SRP.

## 2 Materials and methods

### 2.1 Materials

Fresh *S. rugosoannulata* was purchased from Wuhan Yuanlai Ecological Agriculture Technology Co., Ltd (Wuhan, China). Sodium hydroxide, hydrochloric acid, potassium bromide, and sodium dodecyl sulfate (SDS) were obtained from Sinopharm Chemical Reagent Co., Ltd (Shanghai, China). TGase (BR, 200 u/g) was purchased from Shanghai Yuanye Biotechnology Co. Ltd (Shanghai, China). All other chemical reagents used in the experiment were of analytical grade.

### 2.2 Pre-treatment of *s. Rugosoannulata*

Fresh *S. rugosoannulata* was cleaned and sliced. The drained slices of *S. rugosoannulata* were put into a hot air circulation drying oven, baked at 50℃ for 8 h, crushed through a 100-mesh sieve, to get the sieve material for protein powder, placed in a desiccator, sealed and preserved.

### 2.3 UAE conditions and determination of SRP

Based on the optimal extraction conditions of the traditional alkaline method, and make modifications. Accurately weighed 10 g of *S. rugosoannulata* powder, mixed it thoroughly with water in a ratio of 1:30 g/mL, with 2 mol/L NaOH to adjust the system pH value to 12 for ultrasonic treatment. Ultrasonic parameters: ultrasound temperature of 40℃, ultrasound power (240 W, 360 W, 480 W), ultrasound time (5 min, 10 min, 15 min, 20 min, 25 min, 30 min). After ultrasonic treatment, the sample solution was centrifuged at 8,000 r/min for 15 min, and the supernatant was taken as the crude protein extract. pH of the extract was adjusted to an isoelectric point of 4.2 with 2 mol/L HCl, and then centrifuged at 8,000 r/min for 10 min after standing for 80 min. The precipitate was repeatedly washed and centrifuged, dissolved with a small amount of water, and adjusted to a neutral pH. Then, dialyzed for 24 h under the condition of 4℃ after stirring evenly, and pre-frozen for 12 h under the condition of −20℃. Freeze-drying in a vacuum freeze dryer (LGJ-10, Beijing Song Yuan Huaxing Science and Technology Development Co., Ltd.) for 36 h to prepare SRP powder. The protein powder was prepared and stored at −20℃ for future use. The calculation formula of protein yield is as follows:

$\begin{array}{c} \text{S}\text{R}\text{P y}\text{i}\text{e}\text{l}\text{d}\text{(}\text{\%}\text{)}\;=\;\text{Quality of SRP powder}/\text{Quality of S. rugosoannulata}\text{×}\text{100}\text{\%}\# \end{array}$ (1)

### 2.4 Process optimization of TGase

#### 2.4.1 Single-factor experiment

SRP samples were weighed at 1 g and prepared into an 8 % (w/v) protein suspension. pH was adjusted, TGase was added, and reacted at the setting temperature and time, then the enzyme was inactivated at 90℃ for 5 min. Pre-frozen at −20℃ for 12 h, the sample was then freeze-dried in a vacuum condition for 36 h, and the modified SRP was prepared and stored at −20℃ for future use. Single-factor experiments were carried out to study the effect of reaction pH, cross-linking time, cross-linking temperature, and TGase dosage on the water-holding capacity of SRP. All the experiments were repeated three times.

#### 2.4.2 Response surface methodology

Based on the results of the single-factor experiments, the factors and levels that had an influence on the water-holding capacity of SRP were selected, and SRP water-holding capacity was taken as the response value. The Box-Behnken test was designed by using the Design-Expert 13 software to conduct a three-factor and three-level response surface analysis test for the reaction pH, cross-linking temperature, and TGase dosage. Response surface analysis was used to analyze the interaction between each factor, and the optimal results obtained from the software analysis were verified. The levels of the factors in the response surface test are shown in Table 1.

**Table 1:** Factors and levels of response surface tests.

**Levels**	**pH(A)**	**Temperature/℃(B)**	**TGase dosage/%(C)**
−1	4	30	0.2
0	5	40	0.4
1	6	50	0.6

### 2.5 Zeta potential

Measurements were performed using the nanoparticle size potential analyzer (Nano ZS, Malvern Instruments Ltd, London, UK) with a protein sample solution concentration of 1 mg/mL. The instrument parameters and relevant experimental conditions were as follows: the sample type was protein, the refractive index of the particles was 1.450, the absorption rate of the particles was 0.000, the dispersant was water, the refractive index of the dispersant was 1.330, the absorption rate of the dispersant was 0.8872, the temperature of the test was 25℃, the sample stabilization time was 1 min. The sample cell was transparent on all sides with a light diameter of 1 cm [17].

### 2.6 Fluorescence spectroscopy

A 0.5 mg/mL protein solution was prepared by dissolving powdered SRP in ultrapure water. The protein solution was vortexed and shaken for 2 min, and aspirated into the sample cell. Experimental parameters: excitation wavelength of 280 nm, scanning rate of 20 nm/s, scanning range of 300 nm-400 nm, excitation slit width of 5 nm, emission slit width of 5 nm.

### 2.7 Fourier transform infrared (FTIR) spectroscopy

The freeze-dried SRP was dehydrated at 25℃ for at least 2 h to minimize the effect of residual moisture. Then, the SRP sample was thoroughly ground by adding 500 mg of KBr and placed in a vacuum tablet press and pressurized to 25 MPa for 45 s. The prepared sample presses were placed in a sample chamber and scanned cumulatively 64 times at a frequency of 4000 cm^−1^ to 400 cm^−1^ at a resolution of 4 cm^−1,^ minus the background mapping with KBr presses as blanks.

Spectral analysis: comparing the infrared spectra obtained by scanning, the characteristic band of the protein, the amide Ⅰ band, was selected to be resolved, and the α-helix, β-sheet, β−turn, and random coil content. The spectra were processed using OMNIC software, and then the amide I band was baseline corrected, deconvolution processed, and second-order derivative peak splitting fitted using PeakFit software to obtain the SRP secondary structure content [18].

### 2.8 X-ray diffraction (XRD) analysis

An appropriate amount of SRP sample powder was placed in a desiccator. The parameters of the X-ray diffractometer (D8-ADVANCE, NETZSCH-Gerätebau GmbH, Sable, Germany) and the relevant experimental conditions were as follows: the X-ray tube was of a ceramic type with a Cu target at a wavelength of 0.154 nm, the angle of diffraction (2θ) was 10°-50°, and the scanning speed was 4°/min [19].

### 2.9 Scanning electron microscopy

The method was based on previously reported literature [20], with minor modifications. The SRP powder sample was uniformly coated on the conductive adhesive, and any powder that did not adhere was gently blown off using a wash ball. The dried sample was mounted on a bronze tube for spray gold plating with an ion sputtering apparatus, and the morphology of the samples was observed at magnifications of 200x, 1000x, and 8000x at an accelerating voltage of 10 kV.

### 2.10 Water-holding capacity

Accurately weighed 0.1 g of SRP sample in a 2 mL centrifuge tube, slowly added 1 mL of distilled water, mixed it with sufficient oscillation at room temperature, followed by standing for 20 min to fully absorb water, centrifugation at 4000 r/min for 15 min, and weighing after discarding the supernatant. The water-holding capacity (WHC) of SRP was calculated according to the formula:

$\text{WHC = (}{\text{A}}_{\text{3}}\text{-}{\text{A}}_{\text{2}}\text{)}/{\text{A}}_{\text{1}}$ (2)

Where WHC is water-holding capacity, g/g. A_1_ is the quality of SRP, g. A_2_ is SRP and centrifuge tube quality before centrifugation, g. A_3_ is SRP and centrifuge tube quality after centrifugation, g.

### 2.11 Oil-holding capacity

Accurately weighed 0.1 g of SRP sample in a 10 mL centrifuge tube, slowly added 4 mL of soybean oil, and mixed it with sufficient oscillation at room temperature, followed by standing for 20 min to fully absorb water, centrifugation at 4000 r/min for 15 min, and weighing after discarding the supernatant (free oil). The oil-holding capacity (OHC) of SRP was calculated according to the formula:

$\text{OHC}\;=\text{(}{\text{B}}_{\text{3}}\text{-}{\text{B}}_{\text{2}}\text{)}/{\text{B}}_{\text{1}}$ (3)

Where OHC is oil-holding capacity, g/g. B_1_ is the quality of SRP, g. B_2_ is SRP and centrifuge tube quality before centrifugation, g. B_3_ is SRP and centrifuge tube quality after centrifugation, g.

### 2.12 Emulsifying activity index (EAI) and emulsion stability index (ESI)

The protein was prepared as 1 % (w/v) protein solution, 6 mL of the sample solution was taken, 2 mL of soybean oil was added, the system was vortexed for 2 min to mix well, 50 μL of the bottom emulsion at 0 min and 10 min were taken respectively, 5 mL of 0.1 % (w/v) SDS solution was added, and the absorbance was measured at 500 nm. The EAI and ESI were calculated as follows, respectively:

$\text{EAI (m}\text{2}\text{/g) = (2[MULSGN]2.303[MULSGN]}{\text{A}}_{\text{0}}\text{[MULSGN]n)}/\text{(L[MULSGN]}\rho \text{×}\text{φ}\text{[MULSGN]1000}0\text{)}$ (4)

$\text{ESI (\textbackslash{}\%) =}\;{\text{EAI}}_{\text{10}}/{\text{EAI}}_{\text{0}}\text{[MULSGN]100\%}$ (5)

Where A_0_ is absorbance of protein emulsion at 0 min, L is the width of the colorimetric dish (1 cm), n is the dilution factor with a value of 100, ρ is the protein concentration before emulsion formation, mg/mL, φ refers to the volume fraction of oil phase, 25 %, EAI_0_ is the emulsifying activity index of emulsion at 0 min; EAI_10_ is the emulsifying activity index of emulsion at 10 min [21].

### 2.13 Data statistics and analysis

All experiments were repeated three times, and the experimental data were expressed as means ± standard deviation. The significant differences were analyzed using IBM SPSS Statistics 26, and the differences between groups were analyzed with ANOVA test. The significant differences were expressed as *p* < 0.05. Design Expert 13 was used to design the surface regression equations. Origin 2021 software was used for statistical plotting.

## 3 Results and discussion

### 3.1 The influence of UAE conditions on SRP yield

**Fig. 1:**
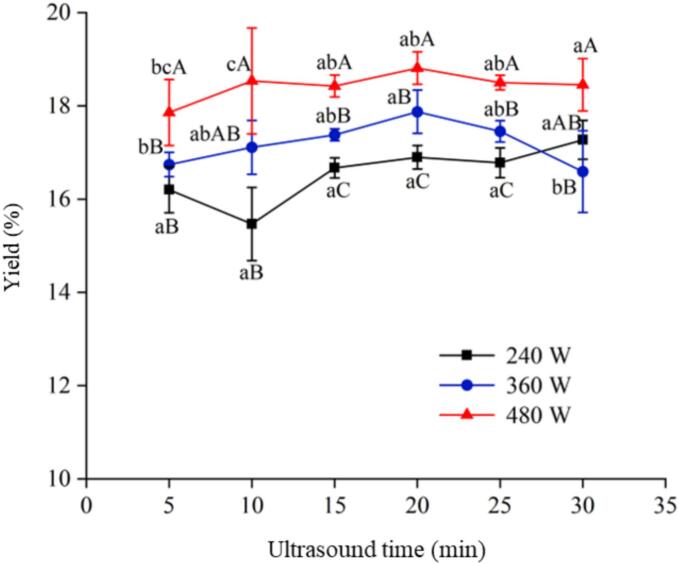
Effects of UAE conditions on SRP yield. Different lowercase and uppercase letters indicate significant differences in SRP under different ultrasound times and ultrasound powers (*p* < 0.05).

As shown in Fig. 1, the yields of the SRP treated with ultrasound were increased. The yield of SRP reached a maximum value of 18.81 % at 480 W in 20 min. The improvement was due to the mechanical effects produced by ultrasound. Ultrasound accelerated the collision between the sample and the solvent, promoted the mutual permeation, and improved the mass transfer rate [22]. In addition, ultrasound could also damage the cell wall of *S. rugosoannulata*, thereby increasing the dissolution of SRP [23].

With the increase of ultrasound power, the yield of SRP increased significantly (*p* < 0.05). When the ultrasound power was 240 W, the power was small, and the ultrasound broke the cell wall of *S. rugosoannulata* weakly, resulting in a lower yield of SRP. When the ultrasound power was increased, the force generated was sufficient to destroy the cell wall, which facilitated the proteolysis. The overall increase in SRP yield with the increase in ultrasound time from 5 min to 20 min might be attributed to the proteolysis of protein molecules, which led to an increase in the SRP yield [24]. The SRP yield decreased after the ultrasound time exceeded 20 min, which was attributed to the fact that the ultrasound destroyed the dissolved proteins. After prolonged ultrasonic treatment, the stomata of the cell wall of *S. rugosoannulata* were further enlarged [25], thus destroying the structure of the SRP and decreasing the amount of SRP dissolved. This finding was consistent with the results reported by Zhao et al. [4] and Badjona et al. [26], as ultrasonic cavitation facilitated mass transfer and reduced extraction time, meaning that continuously increasing ultrasonic extraction time would not enhance protein yield.

### 3.2 The influence of UAE conditions on the fluorescence spectra of SRP

**Fig. 2:**
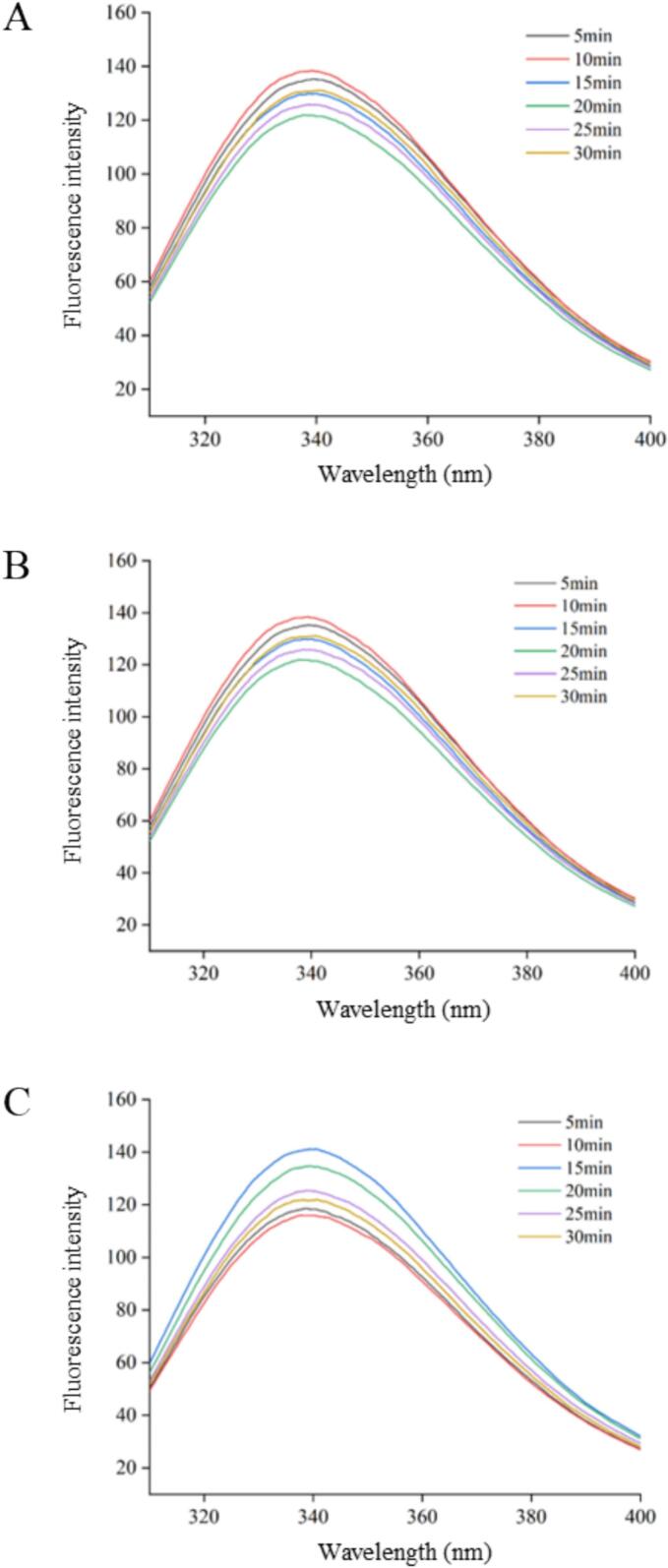
Effects of UAE conditions on the fluorescence spectrum of SRP. (A) ultrasound power 240 W, (B) ultrasound power 240 W, (C) ultrasound power 480 W.

Changes in fluorescence intensity and peak position due to amino acid residues (tryptophan, tyrosine, and phenylalanine) reflect trends in protein conformation and activity [27]. The changes in the tertiary structure of SRP were characterized by fluorescence spectroscopy. As shown in Fig. 2, the intrinsic fluorescence intensity of SRP showed an increasing trend when the ultrasound time was from 0 to 20 min. This may be because ultrasound could generate fluid mixing and shear force through the cavitation effect [28]. High shear force caused the SRP structure to unfold, and the hydrophobic interactions inside the protein molecules were destroyed, exposing more hydrophobic groups or regions within the protein molecules, and the aromatic amino acids were exposed, which led to the enhancement of fluorescence intensity [6]. At 20–30 min, the intrinsic fluorescence intensity of the SRP decreased. The observed fluorescence quenching arose from the weakening of fluorescent molecules by other effects. Under ultrasonic treatment, the protein structure changed. Due to the ultrasonic effect, protein molecules were further stretched, causing cross-linking. Tryptophan, tyrosine, and phenylalanine, which were originally exposed outside the protein molecule, were gradually embedded, leading to an increase in protein steric hindrance and a decrease in fluorescence intensity. As the ultrasound power continued to increase, the fluorescence intensity of SRP also decreased, which may be due to excessive shear force causing some small protein particles to form large protein aggregates again through hydrophobic interactions. Some amino acid residues were exposed, and the interactions were strengthened, leading to protein fluorescence quenching and subsequently reducing the fluorescence intensity [29,30].

In addition, compared with the untreated protein (with a maximum fluorescence emission wavelength of 338.2 nm), the fluorescence spectra of SRP in two treatment groups showed a blue shift: 240 W for 20 min and 480 W for 10 min, while that in the other treatment groups showed a red shift. The protein induced a blue shift after ultrasonic treatment, which may be due to the spatial hindrance between larger molecules weakening the tertiary structure of the protein, or the deamidation of glutamine and asparagine, causing an increase in electrostatic repulsion. Under the treatment conditions of 240 W for 30 min, the maximum fluorescence emission wavelength of SRP increased to 340.2 nm, with a red shift of 2.0 nm. This indicated that residue groups such as tryptophan and tyrosine had undergone changes in both quantity and polarity in their microenvironment. The maximum fluorescence emission wavelength of SRP increased to 340.2 nm under 240 W for 30 min treatment conditions, undergoing a redshift of 2.0 nm. This indicated that the residue groups, such as tryptophan and tyrosine, were altered in number as well as in the polarity state of their respective microenvironments [31]. He et al. [32] proved that proteins had an increase in amino acid residues in polar environments, with differences in maximum absorption peaks attributed to phenylalanine and the aromatic amino acid residues tryptophan and tyrosine. Due to the altered molecular structure of the aromatic amino acids within the protein molecule, its side-chain groups were exposed to aqueous solution, and the ambient polarity gradually increased. The altered SRP fluorescence spectra provided further evidence that ultrasound altered the molecular structure of the proteins [33].

### 3.3 The influence of UAE conditions on the water and oil-holding capacity of SRP

**Fig. 3:**
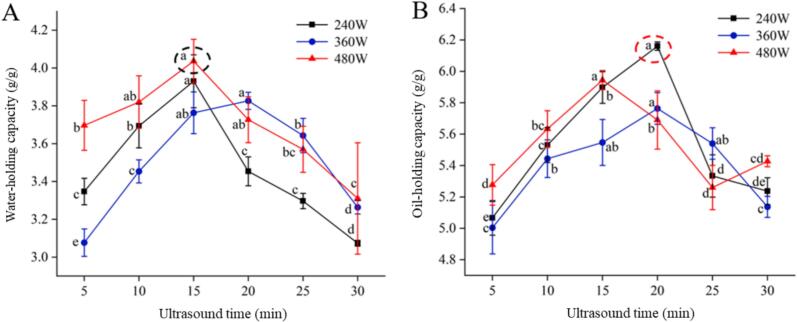
Effects of UAE conditions on water-holding capacity and oil-holding capacity of SRP. (A) water-holding capacity, (B) oil-holding capacity. Different lowercase letters indicate significant differences (*p* < 0.05).

Water-holding capacity refers to the ability of proteins to absorb and retain moisture. The effects of different UAE conditions on the water-holding capacity of SRP are shown in Fig. 3A. The water-holding capacity of SRP reached its maximum value of 4.00 g/g treated by 480 W in 15 min. With the increase of ultrasound time, the water-holding capacity of SRP first significantly increased and then significantly decreased (*p* < 0.05), which was consistent with the trend of SRP fluorescence intensity, probably because ultrasound can lead to a sponge-like structure of the peptide chain skeleton and the formation of some ionized polar groups, which led to the formation of a loose structure and improved the ability to bind water [34]. UAE of *Spirulina platensis* protein also demonstrated that extraction time was a key factor affecting water-holding capacity [35]. With further extension of ultrasound time (20 min-30 min), under strong cavitation, bubble rupture caused by ultrasonic treatment can simultaneously destroy hydrophilic and hydrophobic groups, leading to a decrease in the water-holding capacity of SRP. The formation of high molecular weight protein polymers and aggregates can lead to a decrease in soluble protein concentration, which can also result in poor interactions with water molecules [32].

Oil-holding capacity indicates the ability of the protein to bind with lipids. The effects of different UAE conditions on the oil-holding capacity of SRP are shown in Fig. 3B. With the increase of ultrasound time, the oil-holding capacity of SRP increased and then decreased significantly (*p* < 0.05), reaching a maximum of 6.16 g/g at 240 W for 20 min. Ultrasonic treatment improved the oil-holding capacity of SRP, and the fluorescence chromatograms of SRP showed that ultrasound disrupted the spatial structure of SRP, exposing the internal nonpolar amino acids, and the nonpolar amino acid side chains would bind to more aliphatic hydrocarbon side chains [36]. Moreover, ultrasound denatures proteins, breaking the secondary bonds of the protein molecular structure, unfolding the SRP molecular structure, increasing the protein surface activity, thus enhancing the oil-holding property of SRP [37].

### 3.4 Optimization for TGase modification of SRP

#### 3.4.1 Single-factor experiment

**Fig. 4:**
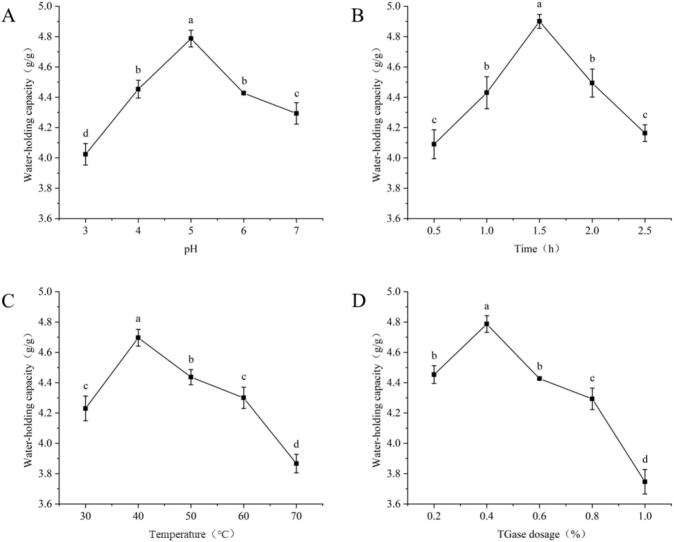
Effects of TGase treatment conditions on the water-holding capacity of SRP. (A) pH, (B) cross-linking time, (C) cross-linking temperature, (D) TGase dosage. Different lowercase letters indicate significant differences (*p* < 0.05).

Fixed reaction conditions: TGase dosage of 0.4 % (w/w), cross-linking time of 1.5 h, cross-linking temperature of 50℃. The effects of reaction pH values of 3, 4, 5, 6, and 7 on the water-holding capacity of SRP were investigated. As shown in Fig. 4A, the water-holding property of SRP increased and then decreased significantly with increasing pH (*p* < 0.05). TGase was reported to improve the structural and functional properties of proteins, and it effectively catalyzed the reaction of lysine and glutamine residues and promoted covalent cross-linking of proteins [38]. At pH 5, the water-holding capacity of SRP reached its maximum, indicating that the environment at this time promoted the cross-linking performance of TGase. The dense and orderly network structure within the sample diminished the degree of freedom of water in the system, improving its water retention and locking capacity. The conformation and activity of enzymes were significantly influenced by the pH value of the solution [39]. As the pH further increased, the water-holding capacity of SRP decreased, possibly due to the low acid concentration reducing the activity of TGase. At pH 3, the water-holding capacity of SRP was the lowest, which was probably because the acidic concentration changed the structure of TGase. Therefore, based on the determination of water-holding capacity, the preliminary analysis showed that the optimal pH is around 5.

Fixed reaction conditions: TGase dosage of 0.4 % (w/w), reaction pH of 5, cross-linking temperature of 50℃. The effects of cross-linking times of 0.5 h, 1.0 h, 1.5 h, 2.0 h, and 2.5 h on the water-holding capacity of SRP were investigated. As shown in Fig. 4B, the water-holding property of SRP was relatively low when the reaction time was 0.5 h. When the time reached 1.5 h, the water-holding capacity was at its maximum. As the cross-linking time further increased, the water-holding capacity of SRP gradually decreased. This was because when the cross-linking time was too short, some of the TGase binding sites of SRP were not fully contacted, which could not be cross-linked with SRP. With the increase of cross-linking time, the active site of TGase was exposed, and the contact with the enzyme became adequate, which promoted the cross-linking action of TGase. At the same time, a network structure was slowly formed between some of the SRP molecules, and the appearance of these pores could strengthen the binding force to water. With the further increase of cross-linking time, the water-holding capacity of SRP showed a decreasing trend, which might be due to the disruption of the stabilizing structure formed, resulting in the weakening of water-holding capacity [17]. Therefore, the optimum cross-linking time was initially analyzed to be around 1.5 h.

Fixed reaction conditions: TGase dosage of 0.4 % (w/w), cross-linking time of 1.5 h, reaction pH of 5. The effects of cross-linking temperatures of 30℃, 40℃, 50℃, 60℃, and 70℃ on the water-holding capacity of SRP were investigated. As shown in Fig. 4C, the water-holding capacity of SRP firstly increased and then decreased within the cross-linking temperature range of 30-70℃, reaching its peak at 40℃. It might be due to the temperature affecting the activity of TGase, thereby affecting the cross-linking effect of TGase. When the cross-linking temperature was kept within the permissible range, the cross-linking effect of TGase was better at 40℃, which led to the exposure of hydrophilic groups inside the protein molecule, strengthening the interaction between the protein molecule and water, and increasing the water-holding property. In addition, research on hemp seed protein indicated that heat treatment could reduce the particle size of the protein, which is helpful for the improvement of water-holding capacity [40]. When the temperature was too high, protein denaturation and cohesion inhibited the interaction between protein molecules and water molecules, and the water-holding property decreased. Therefore, the optimum cross-linking temperature was around 40℃.

Fixed reaction conditions: reaction pH of 5, cross-linking time of 1.5 h, cross-linking temperature of 50℃, investigated the effect of TGase dosage at 0.2 %, 0.4 %, 0.6 %, 0.8 %, and 1.0 % (w/w) on the water-holding capacity of SRP. As shown in Fig. 4D, the water-holding property of SRP increased and then decreased significantly with the increase of TGase dosage (*p* < 0.05). When the TGase dosage was too low, there was still part of the TGase binding site remaining in SRP, and the water-holding property of SRP was low. When the TGase dosage was 0.4 % (w/w), the binding site of SRP was basically saturated, and the water-holding property reached the maximum value. When the TGase dosage continued to increase, the water-holding capacity of SRP had a decreasing trend. This was because the content of SRP was limited; when the TGase sites bound to SRP were fully bound, the excessive TGase could not function properly, and the phenomenon of TGase binding to each other might occur. The excessive enzymes might even compete with each other for the TGase binding sites of SRP, which destroyed the ordered spatial structure of SRP, thus reducing its water-holding capacity [38]. Therefore, the optimum TGase dosage was 0.4 % (w/w).

#### 3.4.2 Response surface test analysis

On the basis of single-factor experiments, the Plackett-Burman (PB) experiment was used to determine the order of the impact of four factors on the water-holding capacity of SRP: reaction pH > cross-linking temperature > TGase dosage > cross-linking time. Therefore, the three factors of reaction pH, cross-linking temperature, and TGase dosage were selected to further optimize the modification process of SRP through a response surface test. The results of the response surface test are shown in Table 2.

**Table 2:** Experimental design and results of response surface.

**No.**	**A**	**B**	**C**	**Water-holding capacity(g/g)**
1	4	40	0.6	4.12
2	5	30	0.2	4.38
3	6	40	0.2	4.63
4	5	40	0.4	4.88
5	6	50	0.4	4.49
6	4	40	0.2	4.29
7	5	40	0.4	4.92
8	6	40	0.6	4.43
9	5	30	0.6	4.24
10	5	40	0.4	4.99
11	4	30	0.4	4.05
12	5	40	0.4	4.86
13	5	40	0.4	4.81
14	5	50	0.2	4.52
15	4	50	0.4	4.25
16	6	30	0.4	4.62
17	5	50	0.6	4.14

Using the software Design-expert 13 for multiple regression fitting, a second-order polynomial equation was obtained for the relationship between SRP water-holding capacity and reaction pH (A), cross-linking temperature (B), and TGase dosage (C): 4.89 + 0.1825A + 0.0138B-0.1112C-0.0825AB-0.0075AC-0.06BC-0.246A^2^-0.2935B^2^-0.2785C^2^.

The results of the response surface test were analyzed by a regression model ANOVA, and the results were shown in Table 3. The response surface regression model was highly significant (*p* < 0.0001). The no significant lack of fit value of the model was 0.733, which was greater than 0.05, and the correlation coefficient of the equations, R^2^, was greater than 0.9, which indicated that the predicted value of the model had a good fit with the experimental value and was relatively reliable [41]. The order of the three factors affecting the water-holding capacity of SRP was reaction pH > TGase dosage > cross-linking temperature.

**Table 3:** Regression model analysis of variance.

	**Sum of squares**	**Degrees of freedom**	**Mean square**	**F-value**	**p-value**	
Model	1.46	9	0.1626	46.63	< 0.0001	significant
A-pH	0.2665	1	0.2665	76.42	< 0.0001	
B-Temperature	0.0015	1	0.0015	0.4338	0.5312	
C-Enzyme dosage	0.099	1	0.099	28.4	0.0011	
AB	0.0272	1	0.0272	7.81	0.0267	
AC	0.0002	1	0.0002	0.0645	0.8068	
BC	0.0144	1	0.0144	4.13	0.0816	
A^2^	0.2548	1	0.2548	73.08	< 0.0001	
B^2^	0.3627	1	0.3627	104.03	< 0.0001	
C^2^	0.3266	1	0.3266	93.67	< 0.0001	
Residual error	0.0244	7	0.0035			
Lack of fit	0.0061	3	0.002	0.4468	0.733	not significant
Pure error	0.0183	4	0.0046			
Total	1.49	16				

**Fig. 5:**
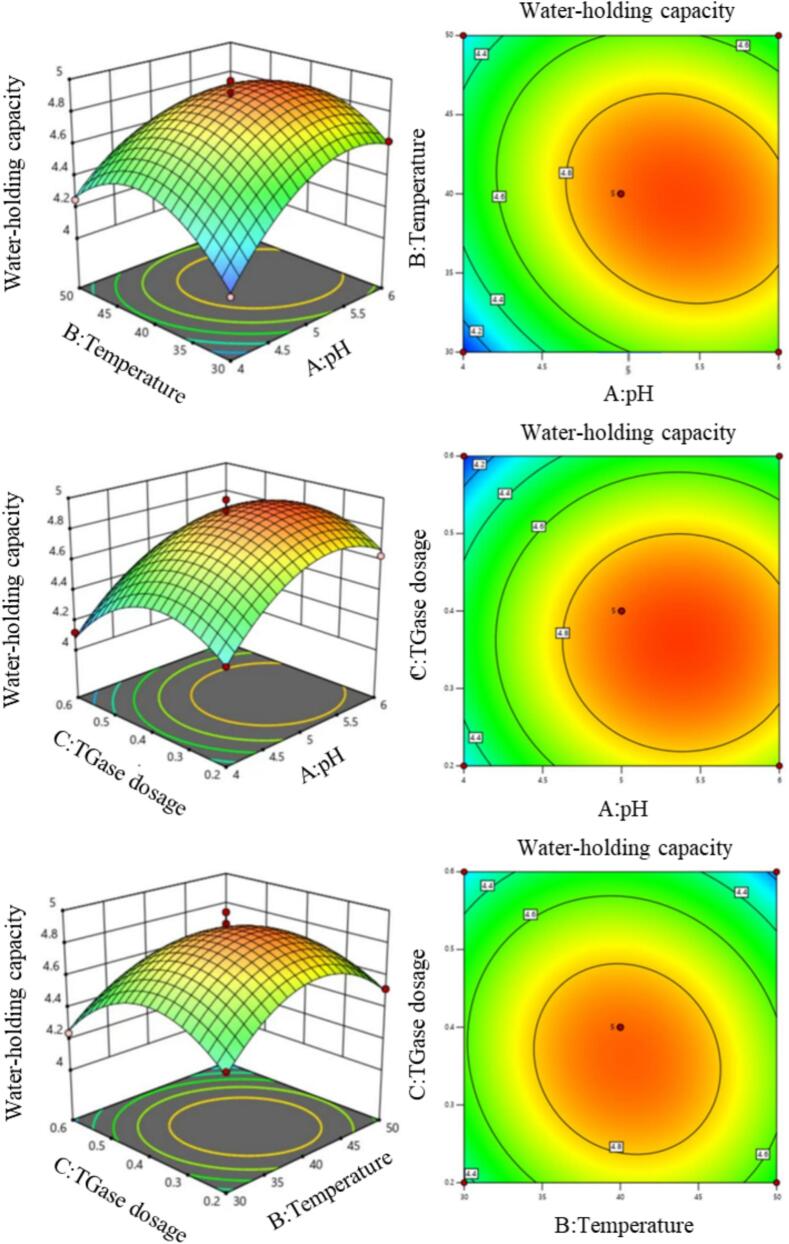
Analysis of response surface interaction.

The results of the two-by-two interaction of the factors on the water-holding capacity of SRP are shown in Fig. 5. The model predicted the optimal process as reaction pH of 5.38, cross-linking temperature of 39.91℃, TGase dosage of 0.36 % (w/w), and the highest water-holding capacity of SRP obtained was 4.94 g/g. In order to obtain a feasible and convenient TGase modification process, the process parameters were adjusted to a reaction pH of 5.4, cross-linking temperature of 40℃, TGase dosage of 0.36 % (w/w) for three validation experiments, and the final water-holding capacity of SRP was 5.02 g/g. The experimental results were fitted with the predictive model.

### 3.5 Potential analysis of modified SRP

**Fig. 6:**
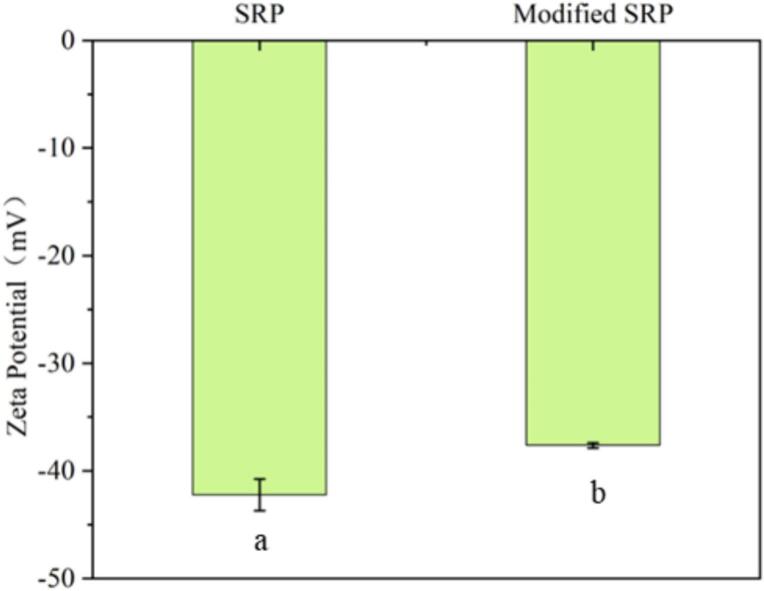
The potentials of SRP and modified SRP.

The zeta potential of proteins serves as a crucial physicochemical indicator, characterizing the surface charge of particles within protein dispersion systems. It is mainly determined by the NH^3+^ and –COO^-^ located on the protein surface and is significantly influenced by the composition of amino acids, protein conformation, as well as environmental factors. Specifically, a higher absolute value of zeta potential indicates a greater surface charge on the particles, ‌and the electrostatic repulsion induced by this charge promotes particle dispersion. A lower absolute value‌ implies ‌a reduced‌ surface charge, which may lead to particle aggregation and precipitation [42]. As shown in Fig. 6, the absolute value of the potential of SRP was 42.23 mV, and that of modified SRP was 37.63 mV. The absolute values of the potentials of both SRP and modified SRP were greater than 30 mV, which indicated the good stability of the two protein suspensions. The greater absolute value of SRP than that of modified SRP indicated strong intermolecular repulsion and less aggregation [43]. This showed that TGase treatment inevitably led to changes in the protein structures. The charged groups in the protein were disrupted, resulting in a decrease in the polarity of SRP protein, thereby reducing the electrostatic repulsion and lowering the potential value of the SRP protein [44].

### 3.6 Particle size analysis of modified SRP

**Fig. 7:**
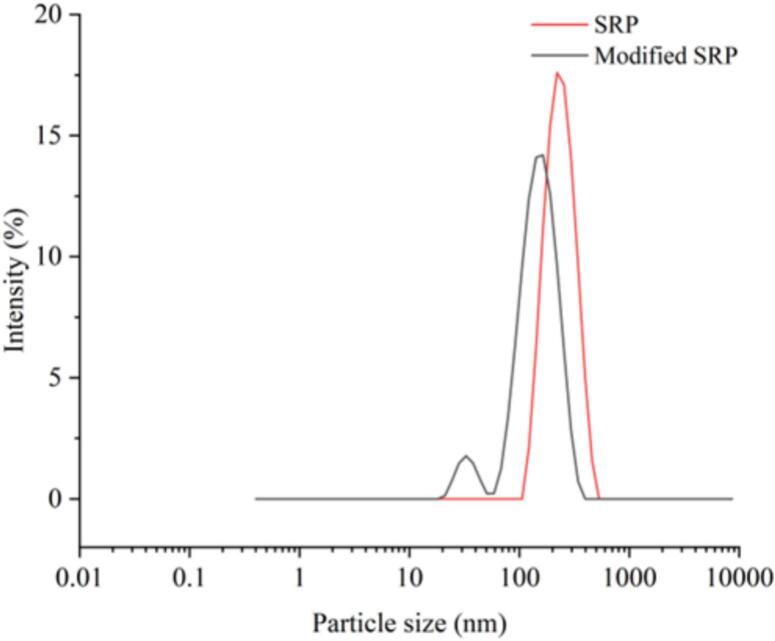
The particle size distribution diagram of SRP and modified SRP.

Protein particle size is one of the main factors affecting its functional properties, possibly by influencing the formation and stability of aggregates in protein solution [44]. The particle size of SRP before and after TGase treatment is shown in Fig. 7. After TGase crosslinking SRP, the particle size distribution of the protein appeared bimodal, indicating the formation of large molecular polymers and some small molecular substances, with particle sizes concentrated in 10 nm-100 nm and 100 nm-1000 nm. Compared with untreated SRP, the average particle size of SRP solution treated with TGase decreased from 192.3 nm to 140.4 nm (*p* < 0.05). Research by Min et al. [45] indicated that the increased number of TGase catalytic sites led to denser protein particles and a corresponding reduction in the particle size of pea isolated protein. Additionally, another possible reason was the restrictive hydrolysis of SRP by TGase, part of SRP was cleaved into small molecular fragments, resulting in a decrease of the particle size of SRP, and thus the distribution of the particle size of the particles was relatively uneven. The TGase treatment destroyed the intermolecular bonds, which reduced the particle size of the SRP. The larger average particle size might be related to the tight structure of the proteins [46].

### 3.7 XRD analysis of modified SRP

**Fig. 8:**
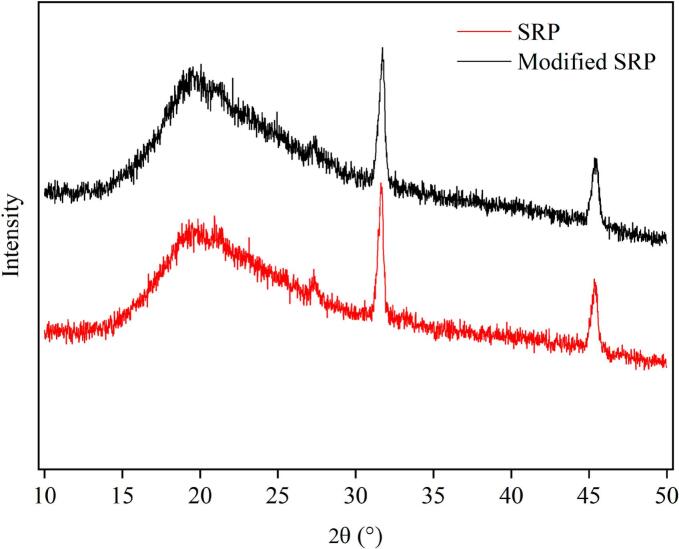
X-ray diffraction patterns of SRP and modified SRP.

To further elucidate the effect of TGase treatment on the structural changes of SRP, the crystal structure of the protein was examined by X-ray diffraction. The diffractograms of SRP and modified SRP were similiar, indicating that TGase did not significantly change the crystal structure of SRP (Fig. 8). Both SRP and modified SRP exhibited a diffuse diffraction peak near 2θ = 19°, while modified SRP showed a broad peak, indicating a change in the crystallinity of the protein, which might be related to the presence of α-helix and β-sheet structure in the protein chain [47]. Ghobadi et al. [48] reported that grass pea (*Lathyrus sativus*) protein isolate showed broad peaks near both 10 and 22, associated with the presence of α-helix and β-sheet in the polypeptide chain structure. A sharp diffraction peak appeared near 2θ = 31° and 45°, respectively, indicating that SRP has a certain degree of crystallinity. The intensity of these sharp characteristic diffraction peaks remained almost unchanged after protein modification, indicating that the crystalline phase of the protein was not disrupted by TGase treatment, which was crucial for the physicochemical stability of the sample [49].

### 3.8 FTIR analysis of modified SRP

**Fig. 9:**
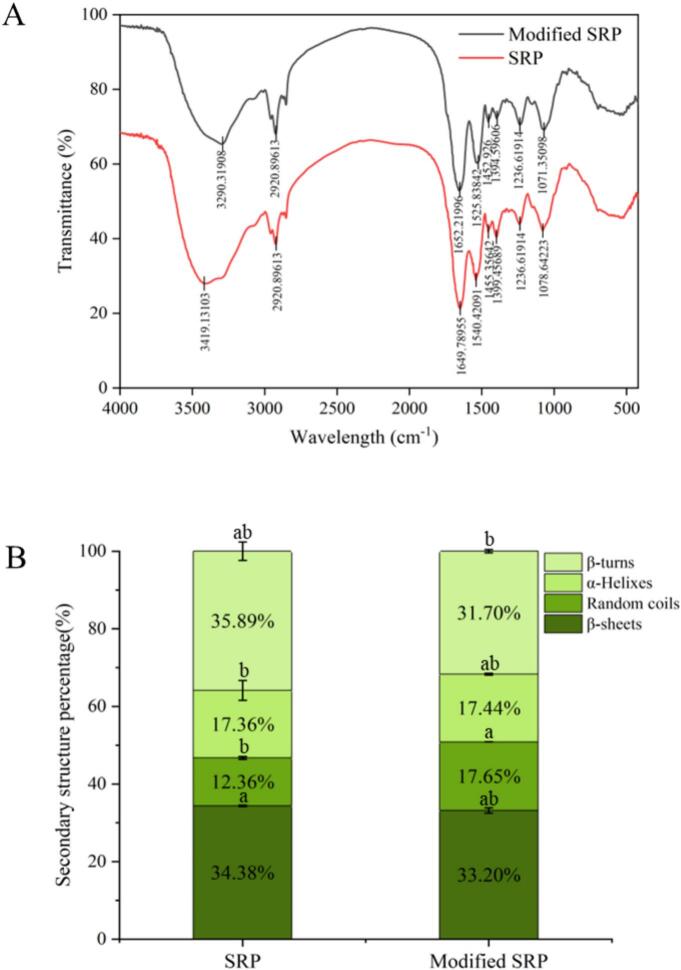
Infrared scanning images(A) and the secondary structure contents(B) of SRP and modified SRP.

Fourier transform infrared spectroscopy was used to analyze the changes in the structure and intermolecular forces between SRP and modified SRP molecules. According to Fig. 9A, the FTIR spectra of the two protein samples showed similar characteristic absorption peaks, indicating that no significant new functional groups were generated during the cross-linking process. The FTIR spectra of unmodified SRP samples showed low absorbance values compared to TGase-treated SRP, and the absorbance values of the main peak of the modified SRP samples increased significantly. The samples had strong broad absorption peaks in the 3200–3600 cm^−1^, wave number region, corresponding to the amide A band. Compared to SRP, the N–H and O–H stretching vibration absorption peaks of modified SRP shifted from 3419 cm^−1^ to 3290 cm^−1^, which was related to the stretching and contracting vibrations of N–H and O–H bonds, and the broadening of the absorption band stemmed from hydrogen bond formation [50]. Upon TGase treatment, covalent crosslinking generated isopeptide bonds. This structural rearrangement raised the numbers of bonded N–H groups, which in turn enhanced the amplitude of amide A [51]. At 2930 cm^−1^ was the C–H stretching vibrational absorption peak. The higher amplitude of this peak could be attributed to hydrogen bonds, protein, and TGase interactions. In addition, the amide I band (–CO-NH-) absorption peak shifted from 1625 cm-1 to 1640 cm-1, and the amide II band (N–H) absorption peak shifted from 1538 cm-1 to 1533 cm-1, mainly characterized by the stretching vibration of the C-O bond, the bending vibration of the N–H bond, and the stretching vibration of the C-N bond [52]. This indicated the secondary structure of the protein has changed. It might be due to the influence of enzyme treatment on the secondary structure, functional groups, and their interactions of the protein molecules [53].

Compared with SRP, the relative content of β-sheet and β-turn decreased, and the relative content of random coil increased in the modified SRP (*p* < 0.05), suggesting that the orderliness of the secondary structure of SRP was disrupted (Fig. 9B). This might be because TGase hindered the increase of β-turn structure by promoting the polymerization of glutamic acid and aspartic acid, the main components of β-turn structure [18]. The transformation of the SRP molecular conformation to a random coil structure confirmed that TGase treatment opened the protein structure and disrupted its intermolecular bonds, causing a certain degree of protein unfolding and leading to a more disordered molecular structure [54]. In addition, the α-helix content was unchanged, probably because the SRP structure was stretched by the extraction temperature and pH, and the hydrogen bond was changed after TGase cross-linking, so that its original α-helix was destroyed, but it still maintained its function [55]. The above results indicated that TGase altered the spatial conformation of the SRP molecule to some extent, resulting in a change in its secondary structure.

### 3.9 Microstructure analysis of modified SRP

**Fig. 10:**
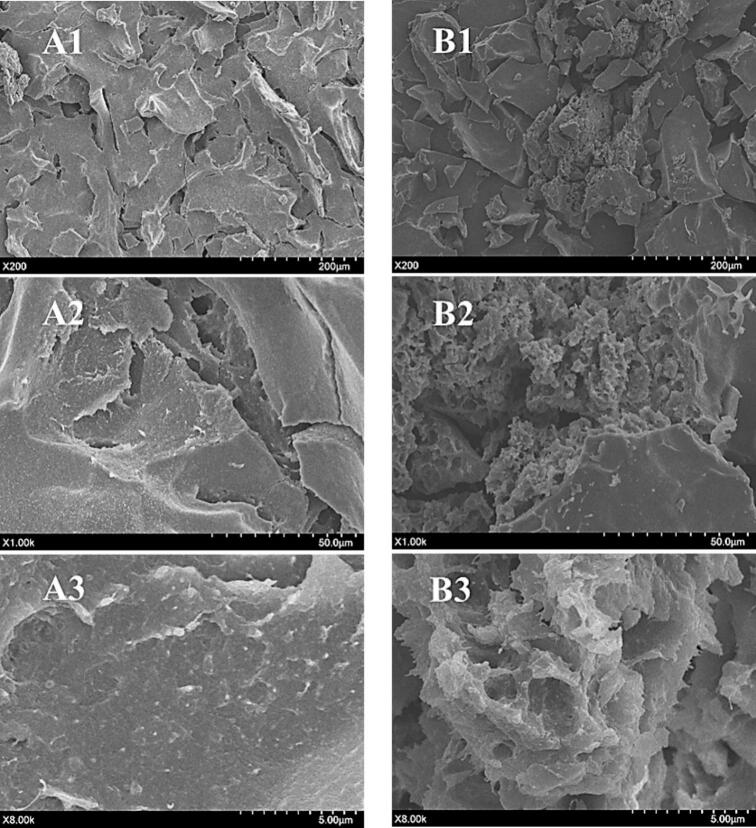
Scanning electron microscope images of SRP and modified SRP. (A1-A3) SRP magnifications of 200 times, 1000 times, and 8000 times. (B1-B3) modified SRP magnifications of 200 times, 1000 times, and 8000 times.

SEM is considered a fast method for analyzing the morphology of protein aggregates, which can display the changes in protein microstructure under different processing conditions [56]. The three-dimensional morphological details of SRP before and after TGase treatment were analyzed at 200, 1000, and 8000 magnifications, and the results are shown in Fig. 10. It was evident that the treatment played a major role in the morphology in the SEM image, and the protein sample with TGase added had a significant difference in morphology from that without TGase. It could be clearly seen that at a magnification of 200, the SRP showed a very regular and dense lamellar structure. After TGase treatment, part of the dense lamellar structure was fractured, producing many small fragments, and the morphology of the sample became looser. The protein structure opened and joined together to form a large whole, indicating that cross-linking of TGase and SRP occurs. Inside, there were many small irregular pores, which were connected, forming a three-dimensional network structure. This network structure was aggregated in some areas, possibly due to insufficient cross-linking reaction between TGase and protein. Previous study had shown that TGase could promote the formation of a three-dimensional network structure of proteins at a certain concentration, which could make proteins aggregate in large quantities and at the same time promote this aggregation, effectively making proteins aggregate in a more orderly way [38]. Based on the results of particle size and spectral analysis, it could be concluded that the addition of TGase caused cross-linking between it and protein molecules, which was beneficial for the formation of protein network structures.

### 3.10 Functional characteristics analysis of modified SRP

The OHC and WHC of protein foods are important indicators in the food industry. EAI and ESI are important indicators of the emulsification properties of proteins, which are key to the role of proteins as food ingredients in food processing. The quality and flavor of food during storage and processing are closely related to the functional properties of proteins. The functional properties of SRP and modified SRP are shown in Table 4.

**Table 4:** Functional characteristics of SRP and modified SRP.

	**WHC (g/g)**	**OHC (g/g)**	**EAI (m^2^/g)**	**ESI (%)**
SRP	4.04 ± 0.16^b^	6.02 ± 0.19^ab^	24.93 ± 2.41^b^	49.17 ± 4.95^a^
Modified SRP	5.04 ± 0.15^a^	6.44 ± 0.08^a^	35.65 ± 3.43^a^	42.43 ± 3.70^ab^

Compared with SRP, the WHC, OHC, and EAI of modified SRP increased, while ESI decreased (*p* < 0.05). According to the results of particle size distribution of SRP and modified SRP, TGase treatment could reduce the average particle size of SRP, and the reduction in particle size could enhance the water-holding capacity of proteins to some extent [57]. The catalytic effect of TGase could cause changes in the secondary and tertiary structure of the protein. As the peptide chain stretches, the exposure of more hydrophobic regions directly increased the OHC of the protein, but the increase was not significant [58]. In addition, TGase caused cross-linking between protein molecules, and the loose and porous structure formed by some small molecule SRP increased the effective surface area of protein in contact with water and oil to a certain extent, which made it easier to solubilize and capture water and oil, thus increasing WHC and OHC. Similar changes were also observed in mung bean protein isolate and cowpea protein isolate [59,60]. This was consistent with the microstructural changes, indicating that TGase could crosslink SRP into large molecules and form a three-dimensional network structure. By modifying protein structure, it further altered its ability to come into contact with water and oil, thereby changing its water and OHC. The ordered structure of proteins tended towards disorder, exposing their hydrophilic groups and hydrophobic side chains, thus making hydrophilic groups more likely to bind with water molecules, while hydrophobic side chains to interact with lipids through the spatial hindrance effect [61]. The EAI of SRP increased, possibly due to the cross-linking effect changed the order of amino acids of SRP, enabling easier formation of protein film between the interface of water and oil, and enhancing the emulsifying ability of SRP. The ESI of SRP decreased, corresponding to the results of zeta potential, probably because the cross-linking of TGase resulted in the loss of charge on the surface of SRP, the repulsion was enhanced, and the formation of the elastic film at the interface of oil–water was blocked [62]. The less net negative charges the SRP molecules carry, the greater the attraction between the droplets forming the emulsion, and the attraction between the droplets was much greater than the repulsion between the droplets, resulting in a less stable emulsion.

## 4 Conclusion

In this work, the effects of UAE and TGase modification on the structure and functions of SRP were investigated. UAE improved the yield of SRP and altered the structure of SRP to increase the water and oil-holding capacity of SRP. TGase treatment caused the SRP to be cleaved into small molecular fragments, resulting in a decrease in protein particle size. The protein conformation was changed, and intermolecular cross-linking occurred. The loose and porous structure in the three-dimensional network of the modified SRP increased the effective area of the protein in contact with water and oil to a certain extent, which improved the WHC and OHC of the protein. Moreover, the cross-linking effect enhanced the emulsification ability of the SRP. The results indicated that UAE and TGase treatment could improve the functional properties of SRP by changing its structure. Therefore, the UAE and TGase modification technology could be used to improve the quality of SRP and enhance its potential as a novel protein in the food industry. Our results would provide information to develop functional foods derived from edible mushroom protein.

## CRediT authorship contribution statement

**Lingyu Yang:** Writing – original draft, Visualization, Investigation, Formal analysis, Data curation, Conceptualization. **Lili Yang:** Investigation, Formal analysis, Data curation, Conceptualization. **Xi Feng:** Investigation, Writing – review & editing. **Yang Xiao:** Resources, Writing – review & editing. **Xiaoju Tian:** Writing – review & editing. **Wen Huang:** Writing – review & editing. **Ying Liu:** Funding acquisition, Project administration, Resources, Supervision, Writing – review & editing.

## Declaration of competing interest

The authors declare that they have no known competing financial interests or personal relationships that could have appeared to influence the work reported in this paper.
